# The Roles of IL-2, IL-7, and IL15 Ligands in B Cells Development from Cord Blood Mononuclear Cells

**Published:** 2015-07-20

**Authors:** Z Aliyari, F Alami, T Mostafavi, H Taiefi Nasrabadi, J Soleimanirad, H Nozad Charoudeh

**Affiliations:** 1 **Stem Cell Research Center, Tabriz University of Medical Sciences, Tabriz, Iran.**; 2 **Student research committee, Faculty of medicine, Tabriz University of Medical Sciences, Tabriz, Iran.**; 3 **Tissue engineering Research group, Novin School of Advanced Medical School, Tabriz University of Medical Sciences, Tabriz, Iran.**; 4 **Tissue engineering group, Novin School of Advanced Medical Sciences, Tabriz University of Medical Sciences, Tabriz, Iran.**

**Keywords:** B cells, CD34+ cells, Cytokine, Umbilical Cord Blood

## Abstract

**Background:**

B cells play an important role in the immune system due to production of the immunoglobulin and secreting several cytokines. It has been shown that B cells produced successfully in the presence of stem cell factor (SCF) and Flt3 ligand (Flt3L). IL2, IL7, and IL15 cytokines as the γc-common family have an essential role in lymphopoiesis. Common γ chain cytokines may support either synergistically or in an additive manner to B cell proliferative activity. Thus, the present study focused specifically on IL2, IL7, and IL15 cytokines that may play a critical role in B cell proliferation in cord blood. In this study, we evaluated the generation of B cells from CD34+/- cord blood mononuclear cells by using IL-2, IL-7, and IL-15.

**Material and Methods:**

Cord blood mononuclear cells were cultured for 21 days in presence of different combination of IL-2, IL-7, and IL-15.Harvested cells were analyzed by flow cytometry at days 0, 7, 14 and, 21.

**Results:**

Present study showed that B cell differentiation from CD34+ cord blood mononuclear cells was increased by using IL-2 and IL-7 at different time points; however, IL7 was more effective (P value < 0.0001). In contrast, IL-15 didn’t increase significantly B cell expansion from CD34+ cord blood mononuclear cell.

**Conclusion:**

These findings showed that IL-2 and IL-7 significantly increased B cell generation from cord blood CD34+ cells; probably this cytokines may be used in ex vivo generation of B cells from cord blood mononuclear cells.

## Introduction

B cells play an important role in the immune system due to production of the immunoglobulin and secreting several cytokines. B-cell population can be divided into several subsets like immature B cells, native B cells, memory B cells and plasma cells which all of them are important in immunological responses (1). B cells develop from hematopoietic stem cells (HSCs) in adult bone marrow (BM) by passing through lineage commitment and several stages before migration to secondary lymphoid tissues (2). It has been shown that CD10+ CD19+ B cells successfully are produced in the presence of stem cell factor (SCF) and Flt3 ligand (Flt3L) from human umbilical cord blood (CB) CD34+ cells within 4 weeks (2). Moreover, human hematopoietic stem cells don’t require the direct interactions with stromal cells for B cell generation (2). IL-7 and Flt3L are known 

to be essential for adult B lymphopoiesis in mice (3,5). In contrast, in human, IL-7 is not required for B cell development (6, 7). However, it has been shown that SCF is important for B cells development (8, 9). In human, Flt3L is critical to cell survival and proliferation of HSPC as well as to B lymphopoiesis (8, 9). It is also reported that IL-7 induces little increase of human B production in co-cultures with stromal cells and B cell proliferate in IL7 independency (6). Moreover, In patients with lack of IL-7 receptor, B lymphopoiesis was normal while T and NK cells development were severely impaired (6,7) .

IL2, IL7, and IL15 cytokines are referred to as the γc-common family (10). The γc protein belongs to hematopoietin receptor superfamily. They play an essential role in lymphopoiesis which its clearly demonstrated by human X-linked severe combined immunodeficiency (11, 12).

IL-2, IL-7, and IL-15 effect on either growth or differentiation of B cells. IL-7, is essential for T cell-independent growth of pre-B cells, while IL-2, and IL-15 influence T cell-dependent B cell proliferation and differentiation (13-16). 

Common γ chain cytokines may support B cell proliferation activity synergistically or in an additive manner. Thus, the present study focused specifically on IL2, IL7, and IL15 cytokines that may play critical role in B cell proliferation in cord blood. It is known that IL-7 has a mutual role in B cell development and NK cells differentiation (17, 18). IL-2 as a T cell growth factor also mediates in activated B cell proliferation and NK cells differentiation (19-21). In cord blood, the B-cell population is enriched (around 11%) as compared to adult peripheral blood (about 5%) (22) . It has been shown that cord blood B cells are able to expressed complete high-affinity IL-2 receptor (1). Umbilical cord blood (UCB) is an alternative source of hematopoietic stem cells (HSCs) for transplantation. B cells are the most important preventive player in post transplantation bacterial infections. Therefore, it is very critical to understand how B cells could be enriched in transplanted cells. 

In this study, the influence of IL2, IL7, and IL15 in the expansion of B cells in CD34+ fraction of cord blood mononuclear cells was evalueted. Furthermore, the most effective cytokine in B cell expansion in cord blood mononuclear cells is investigated. 

## Materials and Methods

***Cell isolation, Culture condition, and cytokines***

Experimental Ethical matters have been approved by Ethical committee of Tabriz medical university. Five cord blood samples were collected from full-term normal deliveries. All samples were diluted 2:1 with phosphate-buffered saline (PBS- SIGM). Then, mononuclear cells were isolated by centrifugation on Ficoll-paque (GE healthcare, 1.078 g/ml) at 850 gm for 25 minutes. The mononuclear cells were collected, washed twice in RPMI1640 (Gibco) and supplemented with 10% FBS (Gibco).

The 10^5^ cord blood mononuclear cells were seeded in 96-well plates in 250 µL of RPMI1640 (Gibco) including 20% fetal bovine serum (FBS; Gibco), and 1% penicillin/streptomycin (Gibco), then supplemented with cytokines including SCF , Flt3 ligand, interleukin 7, IL-15, and IL-2 . All cytokines purchased from PeproTech company (Germany) and used with a final concentration 40 ng/mL. Culture conditions designed in 6 groups including: 1.No cytokine, 2.SCF+Flt3 ligand, 3. SCF+FL+IL-2, 4. SCF+FL+IL-7, 5.SCF+FL+IL-15, and 6. SCF+FL+IL-2+IL-7+IL-15

Cells were incubated at 37°C for 21 days and half of the coculture medium (medium+ cytokines) was replaced at days 7, 14.

***Monoclonal antibodies and flow cytometry***

Monoclonal antibodies used in this study were CD34 (PerCP; clone 581; Abcam) and CD20 (PE; clone 2H7; BD Biosciences). Harvested cells evaluated by flow cytometry at days 0, 7, 14, and 21. We excluded dead cells using Propidium iodide (1.0 mg/mL; Invitrogen). FACS plots were saved by collecting between 10000 to 30000 events and using BD caliber (BD ebioscience). The obtained data were analysed by flowing software (Perttu Terho, version: 2.5.1.). 

***Statistical analysis***

The analysis was performed by GraphPad Prism software (5.04). The statistical significances were determined using one-way ANOVA and Tukey's multiple comparison post-test. P < 0.05 was considered statistically significant. 

## Results

**Role** ***of cytokines in the generation of B cells from cord blood CD34positive and negative cells***

**Figure 1:**
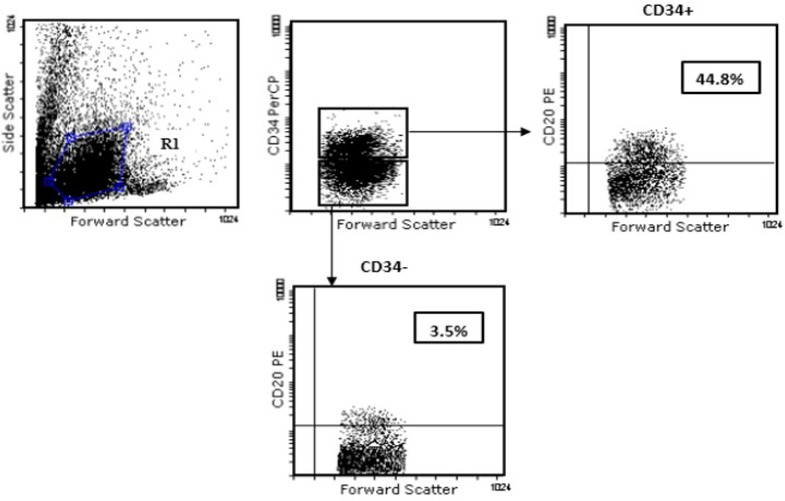
Representative FACS profile of 10^5^ cultured cord blood mononuclear cells in identical time points.CD20^+^CD34^+^ (shown CD34+) and CD20^+^CD34^-^(shown CD34 -) evaluated by gating on lymphoid population in FSC versus SSC.

Harvested cord blood mononuclear cells evaluated by FACS at distinct time periods (Figure1). We gated on CD34+ andCD34- cells to evaluate the percentage of derived CD20+ B cells. As shown in (Figure1), CD34+ cells significantly produced CD20+ cells between 60 to 80 percent more than the CD34- fraction. 

**Figure 2:**
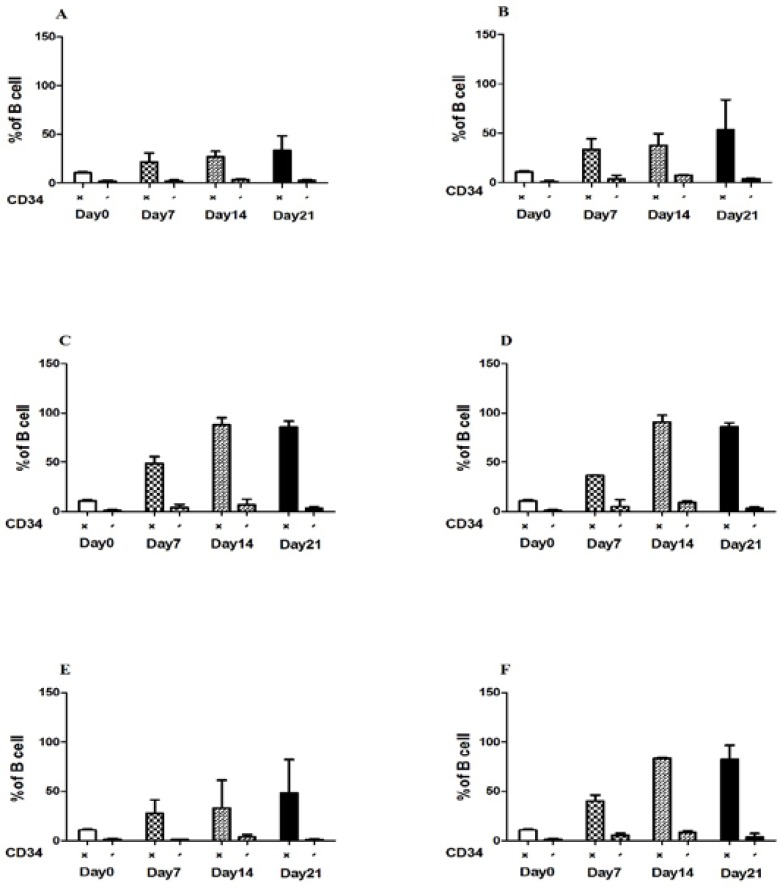
Percentage of CD20+CD34+ and CD20+CD34- cells derived from cord blood mononuclear cells. Flow cytometry and mean (SD) were used to evaluate the expression of CD20+ cells in different time points in the presence of different combination of cytokines: no cytokines (A), SCF+FL(B), SCF+FL+IL-2, P value < 0.0002 (C), SCF+FL+IL-7, P value < 0.0001 (D), SCF+FL+IL-15 (E) and SCF+FL+IL-2+IL-7+IL-15, P value < 0.001(F). The percentage of CD20+CD34+ and CD20+CD34- Cells increased after 14 days of culture in the presence of cytokines. (+) CD34 postitive and (-) CD34 negative

In presence of the combination of all cytokines, the percentage of B cells increased significantly from 21% at day 7 to 90% at day 14 (Figure2). IL-7 in comparison with IL-2 and IL-15 produced higher B cells derived from CD34+ cells (90.65%) (Figure2D). However, on day 21, in the presence of IL-2, the percentage of B cells derived from CD34+ fraction was approximately at same level as for IL-7 (85.7%). However, in presence of IL-2 and IL-15, the percentage of B cells derived from CD34+ cells were 87.8% and 64% respectfully on day 14 (Figure2C, E). Moreover, in combination of all cytokines, the percentage of B cells was 83.3% (Figure2F). There is significant difference between the expansion of CD20+CD34+ and CD20+CD34- in different time periods. In addition, the percentage of CD20+CD34- cells decreased on day21 (Figure2). 

***Mean fleurosence intensity (MFI) of CD20+ cells***

Mean fleurosence intensity (MFI) rate is a numerical data reflecting the severity of antigen expression (23). Mean fleurosence intensity of CD20+ cell evaluated by FACS on day 0, 7, 14, and 21 from cord blood CD34+ cells. 

**Figure 3:**
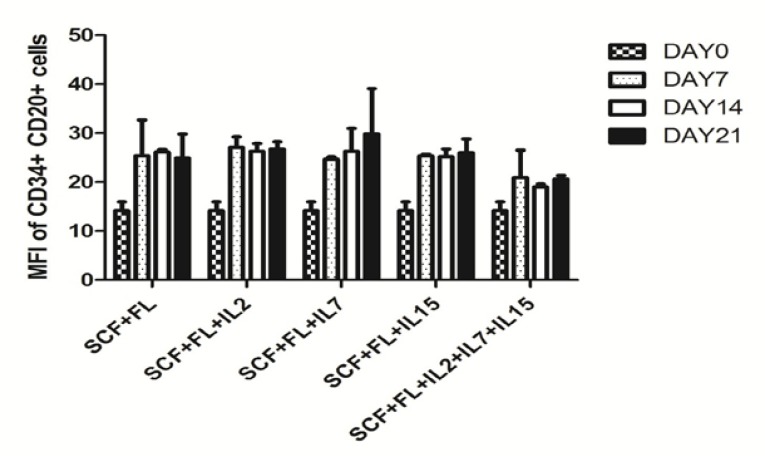
Mean fleurosence intensity (MFI) of different cytokines are in same level. Mean fleurosence intensity of CD20+ cell evaluated by FACS in indicated time points from CD34+ cells derived cord blood mononuclear cells

Our data showed the MFI of CD20+ cells increased from day 0 to day 21 when used SCF+FL +IL7 (P value < 0.004) (Figure3). However, MFI did not change in presence of IL2 and IL15. However, in IL-7 group, MFI increased slightly from day 7 to day 21 and in the last time point, it was higher in comparision to other cytokine combinations. 

## Discussion

Previous studies have shown that IL2, IL7 and IL15 as a member of γ common chain superfamily have critical role in lymphopoiesis. The receptor for each γc-common cytokine may activate more than one intracellular signaling pathway (24, 25). Therefore, B cells proliferation can be affected by cytokines in synergistically or additive way. Several investigators clearly clarified that γc-common cytokines play a critical role in B cell proliferation. In addition, it has been shown that there is a relationship between IL-2 and IL-7 with B cell differentiation (1, 17). B cells derived from cord blood are very important during transplantation for leukemia. Donors are susceptible to infect bacteria and fungi due to suppression of immune system. Therefore, B cell development from cord blood transplanted cells could help donors to recover fast and reduce the relapse in case of bacterial infections. Thus, it is very important to understand whether B cell derived from cord blood cells affected by IL-2, IL-7, and IL-15. In this study, we evaluated the potential of UCB CD34+ cells to differentiate B cells. We cultured cord blood mononuclear cells in the presense of SCF, Flt3, IL-2, IL-7, and IL-15. The percentage of B cells derived from CD34+ and CD34- at different time points was investigated in the current study. Furthermore, the effects of IL-2, IL-7, and IL15 on B cell expansion from cord blood mononuclear cells were explored. The obtained results depicted that IL-7 and IL-2 influenced on B cell differentiation from CD34+ cord blood cells; neverthelse, the effect of IL7 was more than IL-2. As well as in the presence of IL7 the MFI was higher than IL2. In contrast, IL-15 didn’t increase B cell differentiation significantly. Moreover, our data showed that day 14 is the latest time point to get more B cell expansion. This fast differentiation of B cells was only in cord blood cells because they were fast proliferative in comparison with bone marrow. 

## Conclusion

Our data illustrated that IL2 and IL7 can be used in ex vivo expansion to get more B cells from cord blood cells. For further study, it is important primarily to understand the differences between B cell derived by IL2 and IL7and to evaluate the function of derived B cells. 
